# Low-cost, smartphone-based instant three-dimensional registration system for infant functional near-infrared spectroscopy applications

**DOI:** 10.1117/1.NPh.10.4.046601

**Published:** 2023-10-23

**Authors:** Yunjia Xia, Kui Wang, Addison Billing, Matthew Billing, Robert J. Cooper, Hubin Zhao

**Affiliations:** a University College London, HUB of Intelligent Neuro-Engineering, CREATe, Division of Surgery and Interventional Science, Stanmore, United Kingdom; b University College London, DOT-HUB, Department of Medical Physics and Biomedical Engineering, London, United Kingdom; c University of Cambridge, Prediction and Learning Laboratory, Department of Psychology, Cambridge, United Kingdom; d London South Bank University, School of Engineering, London, United Kingdom

**Keywords:** functional near-infrared spectroscopy, diffuse optical tomography, spatial registration, three-dimensional scan, infant

## Abstract

**Significance:**

To effectively apply functional near-infrared spectroscopy (fNIRS)/diffuse optical tomography (DOT) devices, a three-dimensional (3D) model of the position of each optode on a subject’s scalp and the positions of that subject’s cranial landmarks are critical. Obtaining this information accurately in infants, who rarely stop moving, is an ongoing challenge.

**Aim:**

We propose a smartphone-based registration system that can potentially achieve a full-head 3D scan of a 6-month-old infant instantly.

**Approach:**

The proposed system is remotely controlled by a custom-designed Bluetooth controller. The scanned images can either be manually or automatically aligned to generate a 3D head surface model.

**Results:**

A full-head 3D scan of a 6-month-old infant can be achieved within 2 s via this system. In testing on a realistic but static infant head model, the average Euclidean error of optode position using this device was 1.8 mm.

**Conclusions:**

This low-cost 3D registration system therefore has the potential to permit accurate and near-instant fNIRS/DOT spatial registration.

## 1 Introduction

Functional near-infrared spectroscopy (fNIRS) and its extension diffuse optical tomography (DOT) have become noninvasive neuroimaging methods that offer affordable, accessible, and user-friendly solutions for functional brain imaging.^1^ When applying the fNIRS/DOT devices in practice, a three-dimensional (3D) model of the head surface containing the position of the optodes on the scalp is required to ensure proper spatial inferences, a process called “spatial registration.”^2^

The traditional methods for the spatial registration of fNIRS/DOT devices include electromagnetic digitizers^3^^–^^5^ and photogrammetry.^6^^–^^8^ The electromagnetic digitizer method is usually implemented using a stylus to touch the optodes on the device to detect and record their positions. Most electromagnetic digitizers rely on AC magnetic tracking, in which a source produces orthogonal alternating magnetic fields along all three axes. The stylus contains a tri-axis magnetic field sensor that can detect the AC magnetic fields at its location and thus its position relative to the magnetic field source can be determined. However, electromagnetic tracking methods are significantly affected by the presence of metal objects in the surrounding environment,^9^ whereas the acquisition process is also time-consuming. Moreover, this method requires the purchase of a 3D digitizer, which is also relatively expensive (cost ∼£3000).

In contrast, photogrammetry methods are a low-cost solution for spatial registration as they can be easily implemented with a single smartphone.^8^ Photogrammetry takes multiple photographs of the subject wearing the fNIRS device from different angles. The acquired 2D photographs are converted into 3D models (point clouds or meshes) using specialist software (e.g., Metashape^10^). The software analyzes the visual features in the photographs and first estimates the position of the camera associated with each image. By comparing the images and identifying common points and features, photogrammetry software can reconstruct a 3D representation of the object (in our case, the subject’s head). By examining the resulting 3D point cloud or mesh, the optodes’ locations relative to the subject’s cranial landmarks can be determined. However, this process is computationally expensive and time-consuming, thus it is usually performed after the experiment and typically takes many hours with standard computing resources. If the resulting 3D model is not sufficient to capture all the position information of all the optodes, recollecting this information is impossible because the experiment with the subject will have long-since finished.

In addition to the challenges outlined above, both EM tracking and traditional photogrammetry approaches are usually impractical if the subject is an infant, due to their near-constant movement. Given that the head is effectively a rigid object, in theory the movement of an infant subject should not preclude effective photogrammetry. However, in the case of moving infants, traditional photogrammetry approaches face significant challenges. Suboptimal lighting conditions, such as uneven illumination or shadows cast on the infant’s face, can affect the quality and clarity of the acquired images. Additionally, when the subject is in motion, it becomes necessary to mask the background in the resulting 2D images to isolate the infant’s head. These factors collectively make single-camera photogrammetry highly challenging for capturing accurate and reliable 3D head models of moving infants. Recently, a method using the structured-illumination depth camera from a smartphone to acquire a subject’s 3D head model was implemented for spatial registration.^11^ Structured-illumination depth cameras work by projecting specific patterns of light into the field of view and analyzing how these patterns are deformed by the shape of the objects being photographed. Depth cameras can use this information to calculate the distance of each point on the surface of the object from the camera, thereby generating a precise 3D representation of the object.

Compared to the photogrammetry method for fNIRS registration, the directly acquired 3D depth information provided by structured illumination eliminates the time required to convert 2D images into 3D models, potentially allowing the user to adjust the scanning process during the experiment to ensure the model covers all the optodes locations in the scan and are of sufficient quality. Furthermore, by directly acquiring quantified depth information, structured-illumination approaches have the potential to be more accurate and reliable than traditional photogrammetry.

Although this direct 3D scanning method does not require the subject to remain strictly stationary, excessive movement can and will affect the quality of the scanned image. Acquiring a complete 3D model of the head of the moving infant in one acquisition is usually impossible. As a result, when applying the smartphone 3D scanning approach to infants, the user still needs to take multiple snapshots from different angles to produce partial 3D surfaces, and then subsequently stitch them together into a complete full-head 3D model. Although the number of snapshots required is far lower than the number of 2D images required for accurate photogrammetry, this still results in longer acquisition times, reduced accuracy, and prevents instantaneous results.

To overcome these problems, we designed a low-cost 3D scanning device that still leverages the structured-illumination depth camera capabilities but employs a multicamera approach similar to traditional photogrammetry. Our approach uses five older-model iPhones positioned at different angles within a lightweight rig and triggered by a custom Bluetooth controller to yield a whole-head 3D scan in around 2 s. In addition, we propose an alignment algorithm that can automatically stitch the acquired images into a complete 3D head model. The integration of fast full-head 3D scanning and automatic scan stitching significantly reduces both acquisition time and processing time, making it an efficient solution for acquiring comprehensive 3D head scans of infants.

## 2 Methods

### 2.1 System Design

**Fig. 1:**
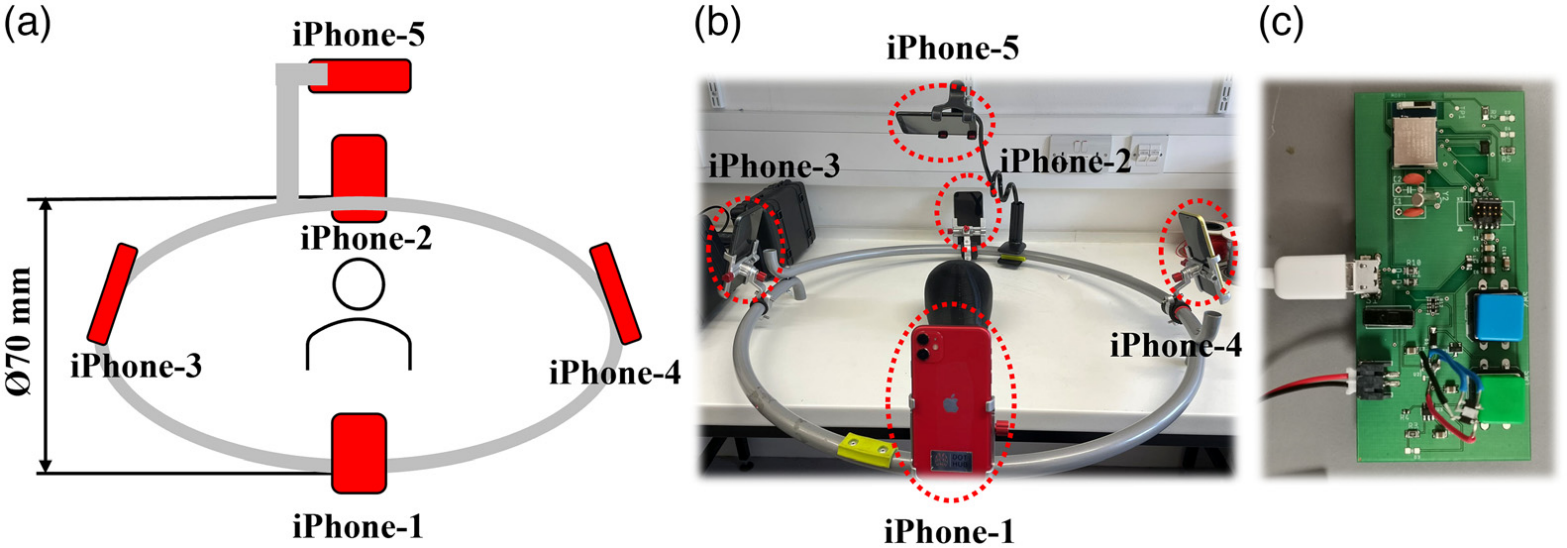
(a) Schematic and (b) the photograph of the proposed system. (c) The designed multichannel Bluetooth controller board.

The schematic and a photograph of the assembled system are shown in Figs. 1(a) and 1(b). Five used iPhone XR smartphones (each purchased for approximately £180) were fixed to a hollow aluminum hoop of diameter ∼70  cm (∼27.6  in.). Four phones were positioned symmetrically around the hoop, with the fifth iPhone fixed by a flexible phone holder ∼35  cm above the plane of the hoop to obtain a scan of the top of the subject’s head. All five iPhone front cameras were oriented toward the center of the hoop. The 3D images of the target were acquired using the “Heges” 3D scanning app.^12^ The Heges app utilized the front-facing structured illumination (“TrueDepth”) camera of the iPhone to directly capture depth-resolved images in a common point-cloud format.

To initiate and control the scanning process of the Heges 3D scanning app on multiple iPhones simultaneously, we developed a custom-designed printed circuit board (PCB) integrated with a multichannel Bluetooth controller and trigger (MDBT50, Raytac, China) [Fig. 1(c)]. The Bluetooth module on our designed PCB board is programmed to send a “volume-down” signal to each of the five iPhones. This signal is recognized by the Heges app as the user pressing a button on the side of the phone to begin image acquisition (functionality that exists in the iPhone and is reminiscent of the shutter release on a traditional camera). After triggering the acquisition, the user partially rotates the hoop 60 deg and then stops the acquisition using the Bluetooth controller. This rotation enhances the coverage and ensures a higher degree of overlap in the acquired point clouds. The increased overlap is essential for achieving accurate point cloud stitching during the subsequent processing. Note that the Heges app was selected from several options available because the developer of the app was willing to add functionality that pushed the resulting images directly from the iPhone to cloud storage without having to actively export the file. This is important to the functionality of our device as it means that the point clouds acquired with all five iPhones immediately and automatically uploaded to the cloud via Wi-Fi to permit instantaneous processing on a local laptop. A Python code running on the laptop downloads the point cloud data from each iPhone using their Internet Protocol addresses, allowing immediate processing to take place.

### 2.2 Infant Registration Model and DOT System

**Fig. 2:**
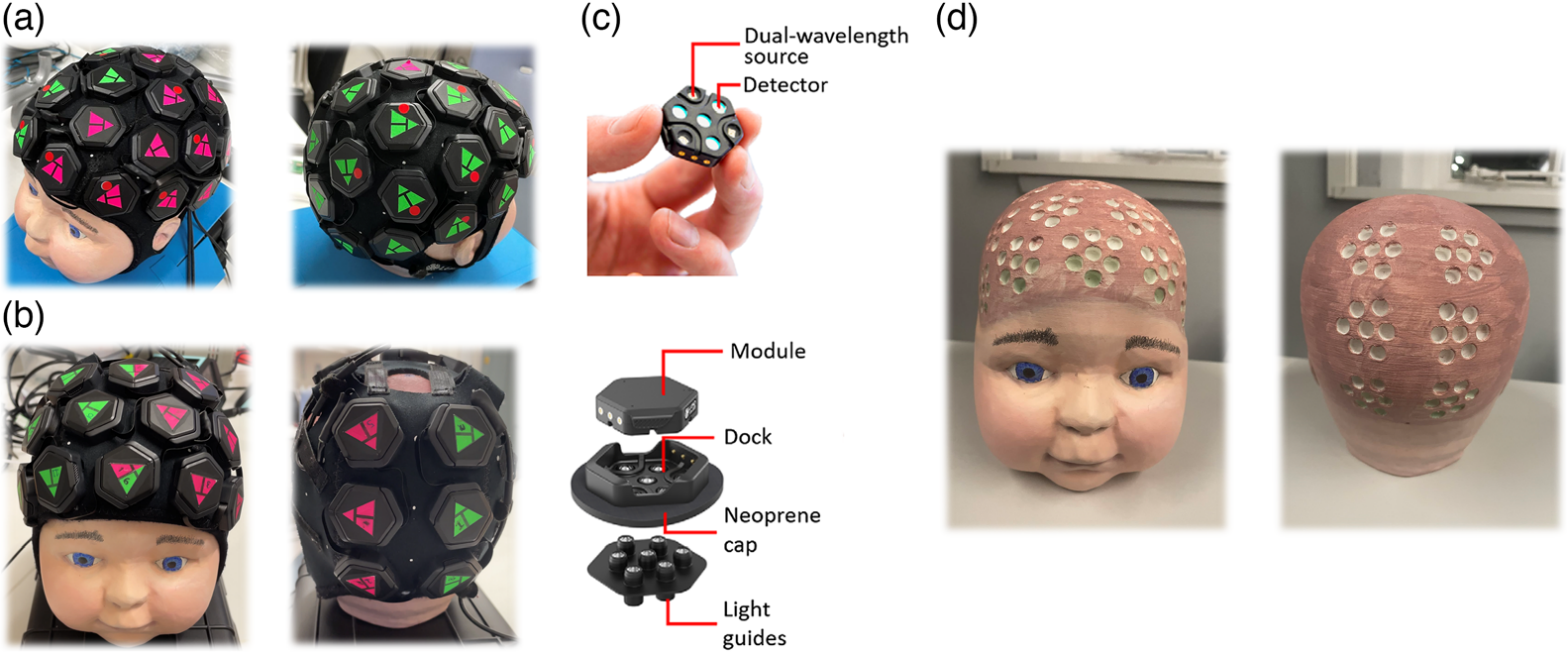
(a) Photograph of the 33-tile and (b) 18-tile LUMO system. (c) The structure of the tile in the LUMO system. (d) The 3D-printed phantom for system validation and evaluation.

Although the methodology proposed here is expected to work for any fNIRS system, it was tested in conjunction with two LUMO high-density DOT (HD-DOT) devices developed by Gowerlabs Ltd.^13^ In the LUMO device, individual hexagonal sensor modules, also referred to as tiles, contain three near-infrared light sources (at 735 and 850 nm) and four optical detectors. This design allows for measurements to be conducted both within each tile and across multiple tiles. By interconnecting multiple tiles through specially designed headgear, users can rapidly construct high-density fNIRS arrays, enabling 3D functional brain imaging. As shown in Figs. 2(a) and 2(b), two devices consisting of 33 and 18 hexagonal modules, respectively, were used to validate the coverage and accuracy of the proposed registration system. The light from the light sources on each LUMO tile is directed to the scalp via short plastic optical fibers called “light-guide” [Fig. 2(c)]. As in the previous applications of the LUMO system, each tile is adorned with a fluorescent green or pink triangular marker on its upper surface, with each corner of the triangle indicating the position directly above one of the tile’s light sources. The triangular marker exists in a flat plane and is precisely positioned, leveraging the fixed and known dimensions of the tile and light guides. Identifying the locations of the three corners of each triangle is sufficient to determine (without approximation) the location of all seven optical contacts to the scalp provided by that tile.

A 3D-printed head model based on the head of a 6-month-old infant was utilized for the validation of the proposed registration system [Fig. 2(d)]. The head model was designed to contain multiple indentations at precisely known positions acquired from the CAD model, into which the light-guides of the LUMO system are to be fitted. As a result, the 3D-printed model allows us to position a LUMO system such that the corners of each triangular sticker (relative to the landmarks of the model) are precisely known. This positioning information can therefore be treated as ground-truth data and used for comparison with the measurements obtained via different positioning methods.

### 2.3 Point Cloud Alignment

As each point cloud was obtained in an arbitrary coordinate system, subsequent processing was required to align and “stitch” the point clouds into a complete 3D head model via appropriate spatial transformations. This alignment can either be performed manually or automatically. For manual alignment, all point clouds were imported into the CloudCompare software. The user manually selected at least three pairs of common points from two meshes, often choosing the vertices of the fluorescent green or pink triangular marker. The software then calculated a rigid transformation matrix that minimized the distance between the point pairs to achieve a global alignment between the meshes. To further enhance the alignment accuracy, an iterative closest point (ICP) algorithm was implemented using CloudCompare. This algorithm facilitates the improvement of general alignment between pairs of point clouds, refining the alignment results achieved through the initial rigid transformation process.

**Fig. 3:**
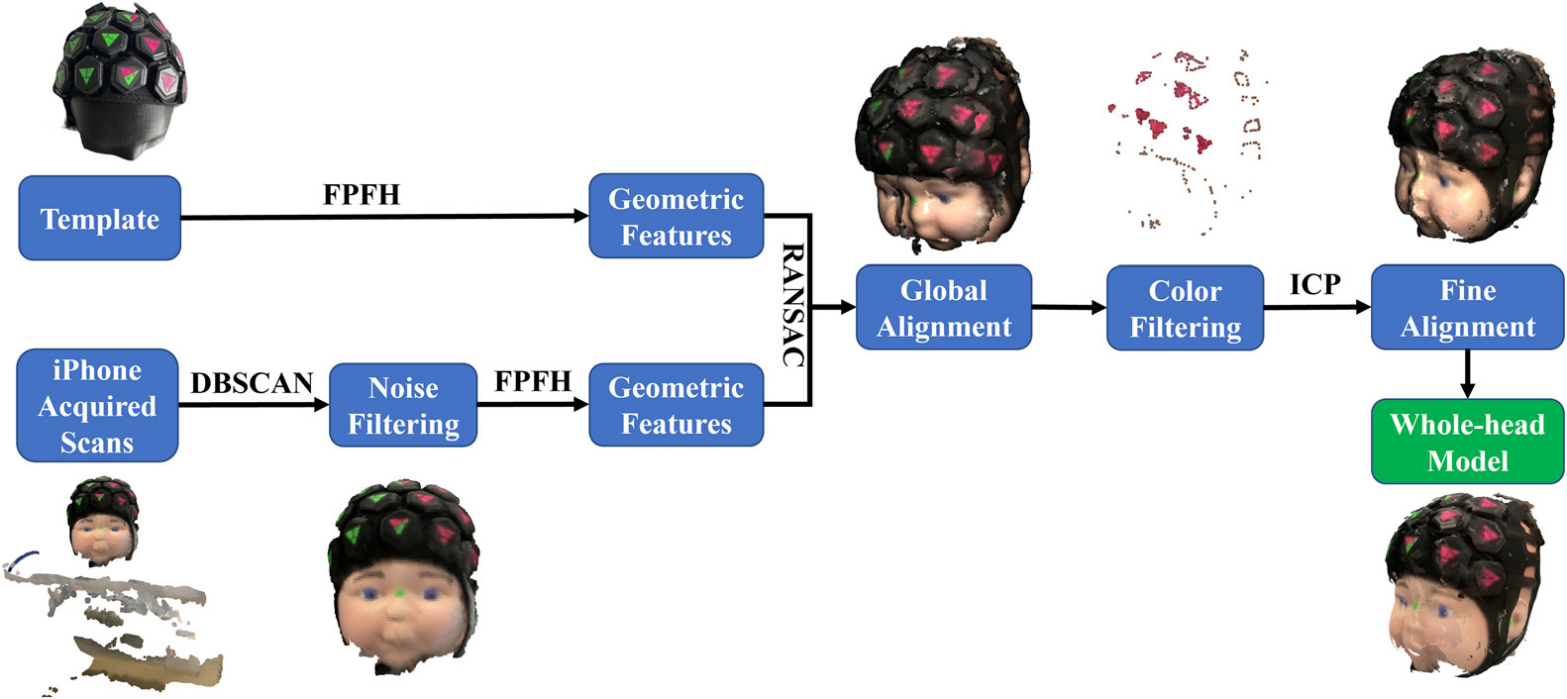
Flowchart of the automatic alignment.

Our automatic alignment pipeline was performed using python with the Open3D^14^ and NumPy^15^ packages. The flowchart for our automatic alignment process is shown in Fig. 3. A density-based spatial clustering of applications with noise (DBSCAN) clustering algorithm was first employed to filter out environmental noise. The DBSCAN algorithm groups points in the point cloud together to form clusters, while labeling outliers as noise. By setting appropriate parameters, this allowed us to effectively remove unwanted noise from the data, ensuring a cleaner point cloud cluster for more accurate alignment. A 3D head template obtained from a previous 3D scan of a different infant head model wearing the same DOT device was used for global alignment. As the spatial features (lines, planes, etc.) of the template phantom wearing the DOT device were similar to that of the target model, a fast point feature histograms (FPFH) vector describing the local geometric properties of each point from the point cloud can be calculated separately for both the template and the scans. A random sample consensus (RANSAC) algorithm was utilized to achieve the global alignment of the scans. In each RANSAC iteration, a user-specified number of points were selected from the scans, whereas the corresponding points in the template point cloud were detected by querying the nearest neighbor in the 33-dimensional FPFH feature space. The point pairs were then used to calculate the transformation matrix for the partial point cloud obtained from each scan. The RANSAC algorithm repeatedly estimates and refines the transformation matrix to find the best alignment that maximizes the number of inlier point pairs and minimizes the effect of outliers. Following the global alignment, the individual scans were color-filtered to keep the green and pink colors corresponding to the triangle markers. This step removed the black background and focused on the relevant features, which were also important spatial features utilized in manual alignment procedure. The color-filtering process improved the accuracy of the alignment. Subsequently, an ICP approach was used to refine the alignment further.

### 2.4 Validation Study

#### 2.4.1 Spatial registration accuracy study on a static phantom

To evaluate the accuracy of the proposed iPhone-based registration system on a static phantom compared with other registration methods, the infant head model and 18-tile LUMO device were used [Fig. 2(b)]. The device has 12 tiles on the front side of the head and 6 tiles on the back side of the head. The cranial landmarks were marked using small (∼4  mm diameter) fluorescent stickers. The phantom was placed in a room with normal lighting conditions, and the proposed registration system method and a standard photogrammetry method were performed. Five individual acquisitions were conducted using the proposed registration system. Each acquisition was performed within ∼2  s.

In the standard photogrammetry method, the user stood in front of the phantom and kept a distance of ∼30  cm between the smartphone camera and the phantom to ensure that the phantom occupies a significant proportion of the field of view. For the first cycle, the smartphone is situated at the phantom’s eye-level and is initially oriented perpendicular to the horizontal plane. During subsequent cycles, the smartphone is lifted and tilted forward by ∼8  cm and ∼20  deg, respectively. This process ensures that the entire phantom, from the neck to the head, is entirely captured on video. The data were recorded using an Apple iPhone 13 Pro, with a camera resolution of 3840×2160  pixels, and a frame rate of 24 frames per second. Twelve frames were selected per second from the video. A total of 635 photos were selected to generate the 3D head model via Agisoft Metashape Standard Edition, version 2.0 (2022).

Once a full 3D model was completed for both the iPhone registration system and the standard photogrammetry approach, the positions of the triangle vertices on each tile indicating the optode positions as well as the cranial landmarks (Nz, Iz, Ar, Al, and Cz) of the phantom from 3D models were manually extracted using CloudCompare.

A standard electromagnetic digitizer approach (Polhemus Patriot, Massachusetts, United States) was also implemented to measure the positions of each triangle’s vertices and the cranial landmarks of the head model.

To allow the accuracy of each approach to be quantified, the positions of the triangular marker vertices obtained from each method were registered to the coordinate space of the CAD model of the head model using a rigid transformation based on the five cranial landmarks. The acquired vertex positions could then be compared to the true positions in the CAD model in which the 3D-printed head model was based. As this registration approach is heavily dependent on the accuracy of the user’s positioning of the cranial landmarks, the Euclidean distance error between the cranial landmarks obtained by each method and the true positions was also calculated to evaluate the accuracy of the positioning of the cranial landmarks in each case.

In addition, to isolate the effect of cranial landmark positioning error, we undertook an alternate approach where the rigid transformation was determined using the vertex points of all the triangular markers. By adopting the alternate approach, we aimed to assess the localization error and its impact on the system’s spatial fidelity during 3D scanning. The Euclidean distance error between the vertex points obtained by each method and the true positions was calculated as the localization error. This evaluation would provide valuable insights into the precision and reliability of the system’s spatial registration and its ability to faithfully reconstruct the spatial features of the scanned subject.

#### 2.4.2 Spatial registration accuracy study on a moving phantom

**Fig. 4:**
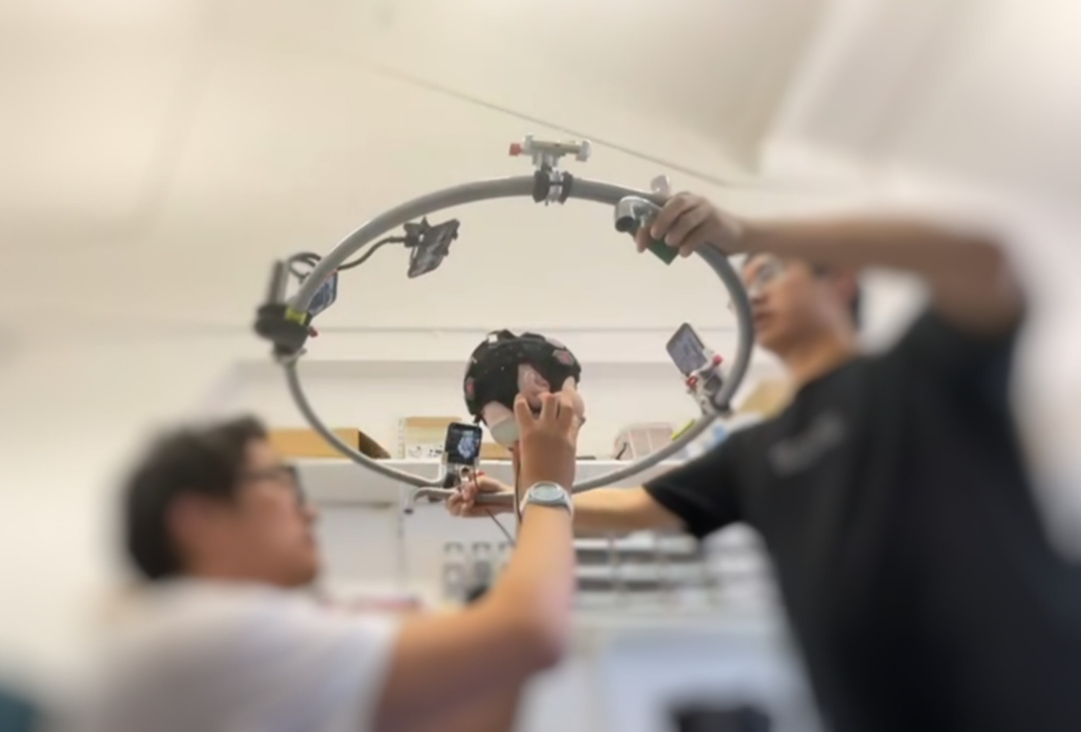
Photograph of the experimenter holding the phantom and mimicking baby head movement.

In the preliminary evaluation of our registration system on dynamic scenarios involving a baby phantom, the 3D printed phantom wearing 18-tile LUMO device was utilized, as detailed in 2.4.1. Prior to scanning, the second experimenter was trained to replicate the movements of the head of a baby based on an open-source video of 6-month-old infants.^16^ Before conducting the scans, the experimenter’s capability to accurately reproduce these motions was verified by comparing the simulated movements with video clips simultaneously, whereas the second experimenter was blinded to the video. The phantom was held by the second experimenter while simulating the head movements of a 6-month-old infant as shown in Fig. 4. The proposed system obtained five acquisitions of the dynamic phantom. These acquisitions were subsequently processed and integrated into one whole-head 3D point cloud of the phantom, utilizing both manual and automatic alignment techniques.

The 3D positions of cranial landmarks and the vertex points of triangle markers on the LUMO devices were extracted using CloudCompare. These positions were then compared with ground-truth data derived from the 3D printed CAD model. Following the evaluation paradigm outlined in 2.4.1, a rigid transformation was performed based on the cranial landmarks between the manually/automatically merged scan acquired by system and ground-truth data. The Euclidean distance error between the vertex points obtained by the proposed system with two different alignment methods and the true positions was calculated as the localization error.

Furthermore, to isolate the influence of the cranial landmark positioning error, another rigid transformation based on the vertices of the triangle markers on LUMO was implemented. The Euclidean distance error between the vertex points obtained by the proposed system with two different alignment methods and the ground-truth positions was calculated as the localization error after isolating the effect of cranial landmark positioning error.

#### 2.4.3 Scan coverage

To evaluate the proportion of the head surface that could be captured by the proposed registration system, the realistic infant head model was used in conjunction with a whole-head, 33-tile LUMO cap [Fig. 2(a)]. To a good approximation, these 33 tiles cover the whole of the accessible scalp of a 6-month-old infant head model. The proposed registration system was used to acquire five scans of the infant head model to evaluate the consistency of the system scan coverage. For the 33-tile LUMO device, a total of 99 triangle vertices and five cranial landmarks of the phantom should be covered. In the resulting aligned 3D scans, blurred and missing triangle vertices were considered to have not been covered by the device. The number of clearly identifiable triangular vertices in the 3D models was counted to evaluate relative scan coverage.

## 3 Results

### 3.1 Localization Accuracy Study on the Static Phantom

The localization error of marker vertices and cranial landmarks was determined using the proposed registration system. The marker vertices indicate the position of the optodes on the DOT device, while cranial landmarks are distinctive points on the skull. The average Euclidean distance error between the position obtained using the registration system and the true value was calculated for each marker vertex or cranial landmark in five acquisitions. These marker vertices and cranial landmarks play a crucial role in spatial registration.

**Fig. 5:**
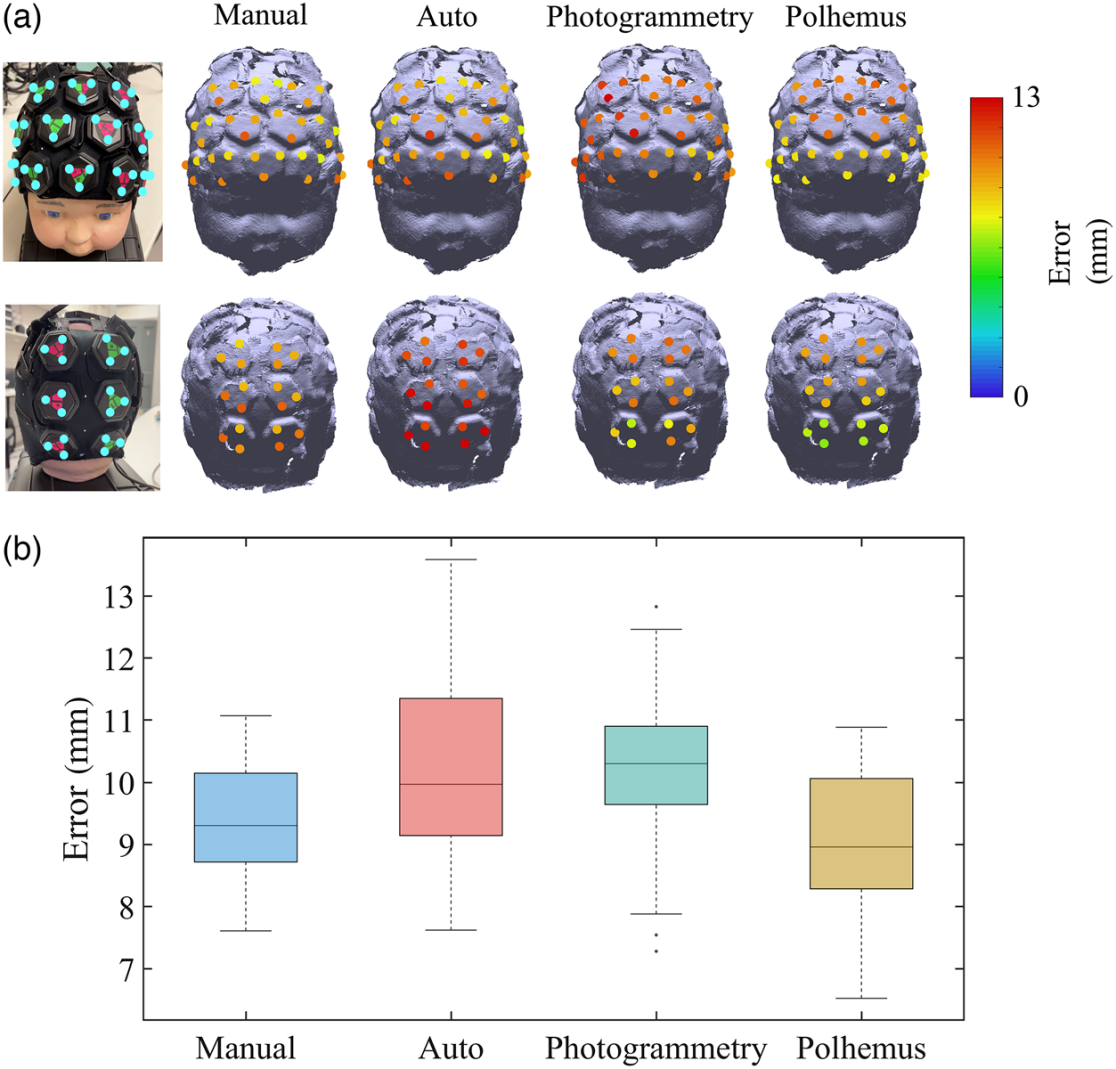
(a) Localization errors of all marker vertices acquired via cranial landmark-based registration using each employed method including the registration system with manual alignment and with automatic alignment, standard photogrammetry, and Polhemus. Localization errors are represented with a color code on the phantom model. Each value indicates the Euclidean distance between the marker vertex on the original phantom and the node on the model acquired with each method. (b) Boxplots represent the distribution of the errors across all marker vertices after the spatial registration process, separately for each employed method. The box’s bottom and top edges represent the 25th and 75th percentiles, respectively, while the central mark represents the median. Outliers are illustrated as dots.

The median localization errors of each marker vertices acquired via the three different methods were calculated, as determined via cranial landmark-based registration (the approach most representative of a real-world application) are shown in Fig. 5(a). A box plot illustrating the distribution of localization errors across all marker vertices after the spatial registration process is shown in Fig. 5(b). The median localization errors of all marker vertices for the registration system with manual alignment (Manual) and automatic alignment (Auto), standard photogrammetry (Photogrammetry), and Polhemus were 9.3, 10.0, 8.7, and 9.0 mm, respectively. These errors are within the range typically observed in studies using similar spatial registration methods.

**Fig. 6:**
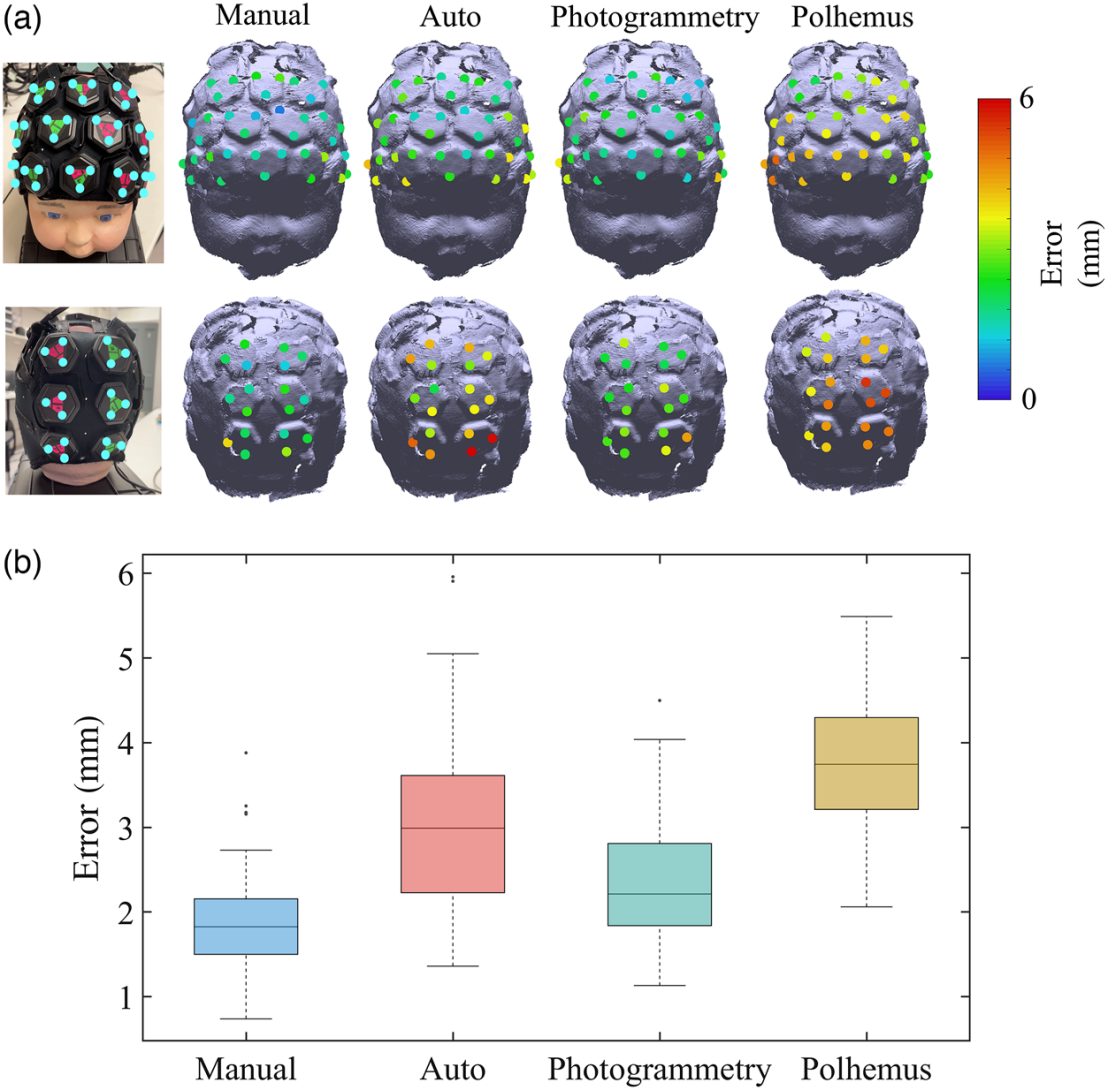
(a) Localization errors of all marker vertices using each employed method after isolating the effect of cranial landmark positioning error. Localization errors are represented with a color code on the phantom model. Each value indicates the Euclidean distance between the marker vertex on the original phantom and the node on the model acquired with each method. (b) Boxplots represent the distribution of the errors across all marker vertices after isolating the effect of cranial landmark positioning error, separately for each employed method. The box’s bottom and top edges represent the 25th and 75th percentiles, respectively, while the central mark represents the median. Outliers are illustrated as dots.

The median Euclidean distance error between the true positions and the cranial landmarks obtained by the proposed registration system with manual and automatic alignment, standard photogrammetry, and Polhemus were, respectively, 10.0, 11.5, 10.9, and 8.2 mm. Moreover, a rigid transformation matrix was calculated using the vertices of all the triangular markers. This approach eliminated the potential localization error caused by user’s inaccurate cranial landmark poisoning. After isolating the effect of cranial landmark positioning error, the median localization errors of all marker vertices for the registration system with manual alignment (Manual) and automatic alignment (Auto), standard photogrammetry (Photogrammetry), and Polhemus were 1.8, 3.0, 2.2, and 3.7, mm respectively. The localization errors across all marker vertices and a box plot illustrating the distribution of the errors after isolating the effect of cranial landmark positioning error are shown in Figs. 6(a) and 6(b).

### 3.2 Localization Accuracy Study on the Moving Phantom

**Fig. 7:**
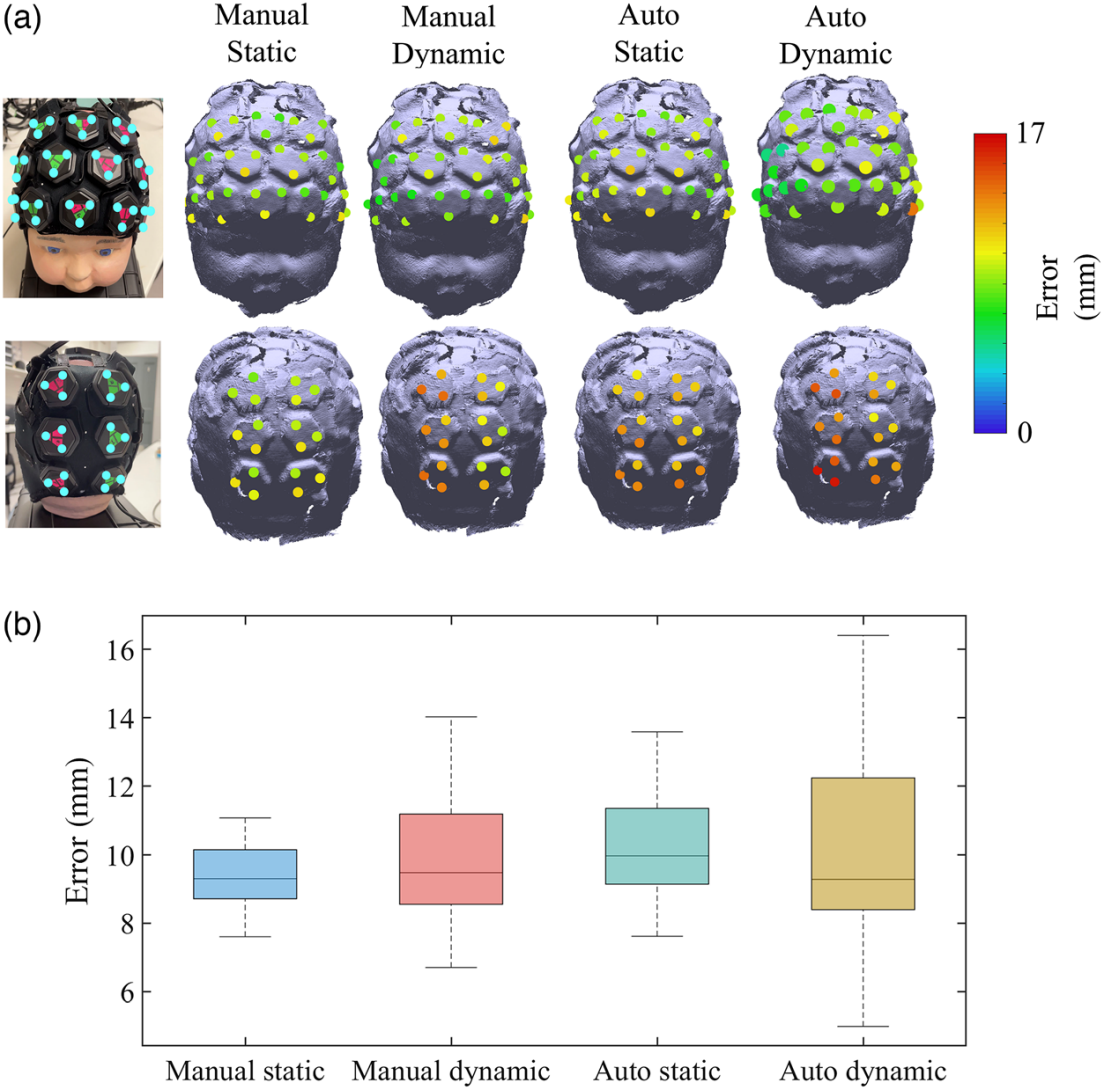
(a) Localization errors of all marker vertices acquired via cranial landmark-based registration using the proposed system, with two alignment methods under static and dynamic phantom. Localization errors are represented with a color code on the phantom model. Each value indicates the Euclidean distance between the marker vertex on the original phantom and the node on the model acquired with each method. (b) Boxplots represent the distribution of the errors across all marker vertices after the spatial registration process, separately for each situation. The box’s bottom and top edges represent the 25th and 75th percentiles, respectively, while the central mark represents the median. Outliers are illustrated as dots.

**Fig. 8:**
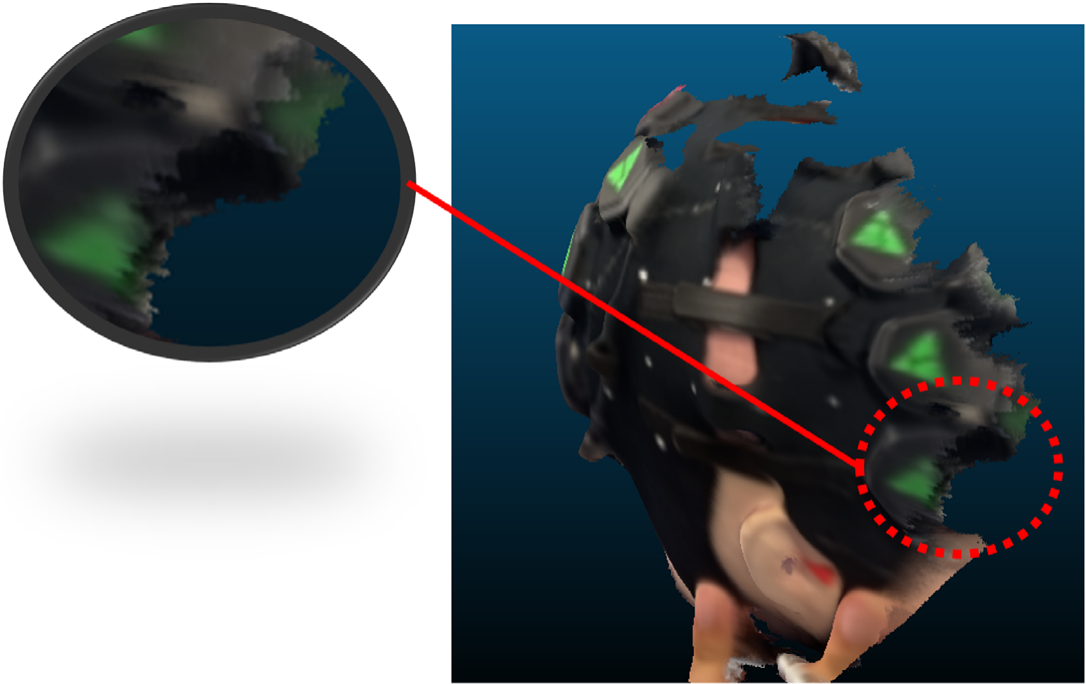
Screenshot of the acquired 3D head model. The subfigure illustrates the severely blurred triangle vertices due to the phantom movement.

The median localization errors of all marker vertices acquired via the system with two different alignment methods were calculated, as determined via cranial landmark-based registration. The localizations errors of the dynamic phantom were compared with the static phantom as shown in Fig. 7(a). A box plot illustrating the distribution of localization errors across all marker vertices after the spatial registration process is shown in Fig. 7(b). The median localization errors of all marker vertices for the registration system with manual alignment and automatic alignment under dynamic phantom situation were 9.5 and 9.3 mm, respectively. In contrast to the static phantom scenario, the movement of the phantom had a relatively minor impact on the localization errors. However, it did lead to an increased occurrence of outliers at the junctions between the scanned images from different smartphones. This is due to the more severe blurring at the edges of the scanned image caused by the phantom movement, as shown in Fig. 8.

**Fig. 9:**
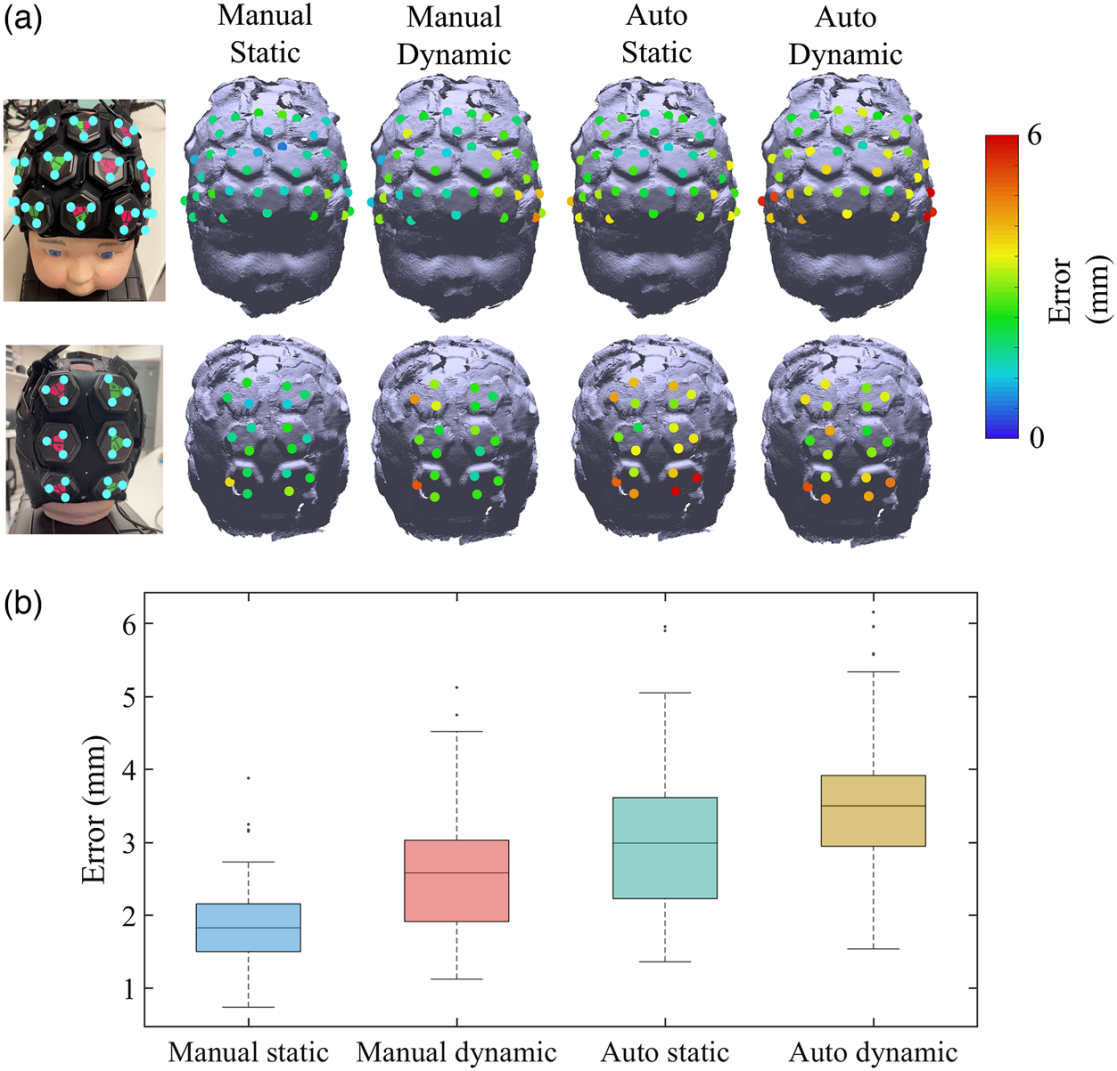
(a) Localization errors of all marker vertices acquired using the proposed system after isolating cranial landmark positioning error, with two alignment methods under static and dynamic phantom. Localization errors are represented with a color code on the phantom model. Each value indicates the Euclidean distance between the marker vertex on the original phantom and the node on the model acquired with each method. (b) Boxplots represent the distribution of the errors across all marker vertices after cranial landmark positioning error isolation, separately for each situation (static/moving phantom). The box’s bottom and top edges represent the 25th and 75th percentiles, respectively, while the central mark represents the median. Outliers are illustrated as dots.

After isolating the influence of the cranial landmark positioning error, the median localization errors of all marker vertices acquired via the system with two different alignment methods were calculated. The localization errors of the dynamic phantom were compared with the static phantom as shown in Fig. 9(a). A box plot illustrating the distribution of localization errors across all marker vertices after the spatial registration process is shown in Fig. 9(b). The median localization errors of all marker vertices for the registration system with manual alignment and automatic alignment under dynamic phantom situation were 2.6 and 3.5 mm, respectively, after the cranial landmark positioning error isolation. The movement of the phantom indeed resulted in an increase of localization errors across all vertices of the triangle marker, particularly in the context of the manual alignment method. In addition, the phantom’s movement also contributed to an elevation in the occurrence of outliers at the junctions between scanned images obtained from different smartphones, which is also due to the significant blurring at the edges of the smartphone scans.

### 3.3 Scan Coverage Study

**Fig. 10:**
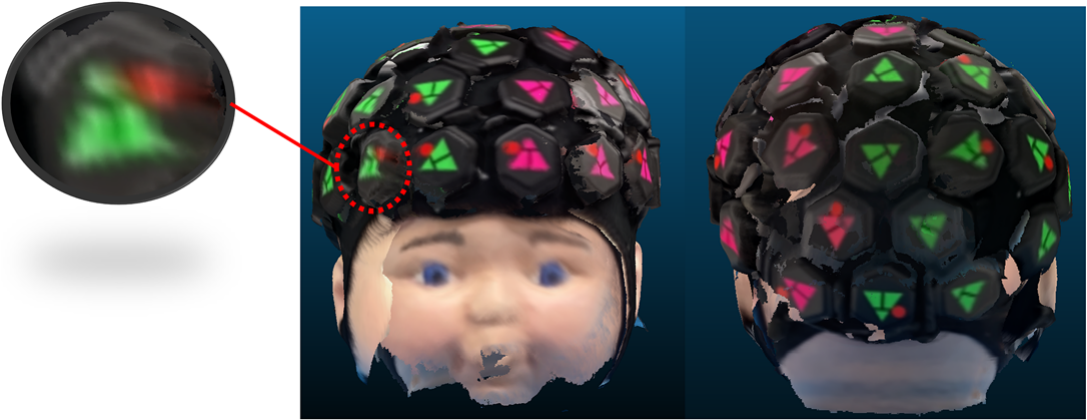
Screenshot of the acquired 3D head model. The subfigure illustrates the blurred triangle vertices. These blurred vertices usually appear at the margin of a single iPhone scan.

The aligned whole-head 3D model of the phantom acquired from one of the scans using the proposed registration system was shown in Fig. 10. As the vertices are indicators of the position of the source and detector on the LUMO device, the number of vertices covered by the registration system relative to the total number of vertices on the LUMO device was calculated as the coverage rate, indicating its ability to cover the positions of the sources and detector as a registration system. No vertices were missing in any acquisitions, and some vertices at the margins of the scanned point cloud were blurred. An average of 0.6 vertices was blurred (3, 1, 1, 0, and 0, respectively), indicating that the proposed registration system was able to capture nearly all (99.4%) of the target’s relevant anatomical features.

## 4 Discussion and Conclusion

The position of each optode on a subject’s scalp and the subject’s cranial landmark is needed when applying fNIRS devices as it is extremely important for appropriate spatial inference. However, traditional methods, such as a 3D digitizer and standard photogrammetry, can be very difficult and time-consuming to implement in infant studies due to the constant movement of the infant.

In this technical note, we proposed a smartphone-based registration system that utilizes the depth camera to directly capture details of the head surface in 3D. Using our custom-designed control board and five iPhone depth cameras fixed on a handheld rig, five 3D scans covering different angles of the subject can be acquired within ∼2  s. The five individual scans can be either manually aligned or automatically aligned into a whole-head model using our alignment algorithm. A 3D-printed phantom based on a 6-month-old infant’s head was utilized for system validation and performance evaluation.

In terms of scan coverage, our result indicates that the proposed system can cover 99.4% of the phantom’s surface location information for a full-head HD-DOT device. In terms of spatial registration accuracy, the result acquired by our system within only 2 s has comparable accuracy performance to that of the standard photogrammetry method and 3D digitizer method, which take much longer time to perform. Moreover, after isolating the cranial landmark positioning error, the localization error acquired by our system with a manual alignment scheme was lower than both 3D digitizer and standard photogrammetry methods, indicating that directly capturing the depth information of the subject has the potential to be more accurate than traditional localization approaches. The localization error of the model obtained by autoalignment was larger than that of photogrammetry, but also smaller than that of Polhemus. This shows that the automatic alignment algorithm we designed has a relatively good performance but can still be further improved.

It is worth mentioning that the ground-truth position information of the phantom was obtained based on the 3D-printed CAD model, should therefore have an accuracy of well below 1 mm, and should therefore not contribute significantly to the measured errors quoted here. Moreover, our proposed automatic alignment algorithm is based on the presumption that the fNIRS device has distinct color characteristics, such as the colored marker on the device. Therefore, the automatic alignment may perform differently for devices with less distinct color characteristics. However, the results presented here give us confidence that via the adaptation of different devices with highly visible markers of known dimensions, and perhaps with small modifications to our alignment processes, our results will be applicable to other devices and configurations.

The moving phantom experiment revealed that the movement of the phantom does introduce a certain level of error increase, primarily due to the highly blurred edge in the smartphone scanned image. However, this increase in error is relatively modest compared to the impact of cranial landmark positioning errors. This contrast underscores the significance of accurately positioning the cranial landmarks of the subject, as it holds greater importance in enhancing the overall accuracy of the system’s registration.

It is important to note that all the results presented here are based on phantoms. As such, there are potential limitations to the proposed system that necessitate additional validation studies. For instance, when applying the system to an actual baby, more intense movements could occur, resulting in increased blurring or even distortion of the scanned images. This can impact the accuracy of alignment for both manual and automatic alignment. Moreover, the layout of the system, with a 70 cm diameter surrounding the subject, may require the baby to be seated either by themselves or with the assistance of a special seat support. Additionally, the potential for distractions to the infant caused by the device remains uncertain. Thus robust assessment of the impact of infant movement on the proposed scanning method and all the comparators (standard photogrammetry, Polhemus measurements, etc.) over a large cohort of infants is required to fully determine the real-world utility of the methodology proposed in this technical note. However, although further refinement and real-world testing and validation are required, we believe that this registration system has the potential to provide an efficient, accurate, practical, low-cost, and universal approach to fNIRS registration.

In terms of future work, beyond conducting validation experiments on real infants, we aim to explore opportunities for system improvements and enhanced compatibility with various devices. One potential solution is to investigate the feasibility of utilizing independent depth cameras in conjunction with open-source computer vision tools. This approach may lead to enhanced system performance and broaden the system compatibility. Additionally, we plan to explore the possibility of using Wi-Fi for triggering scans and previewing depth camera images during the scanning process. This exploration will help determine whether a completely Wi-Fi-based communication approach can further improve the system’s efficiency and functionality. By pursuing these avenues of research, we hope to enhance the robustness and versatility of our proposed system for 3D head scanning, ultimately leading to more accurate and comprehensive results in real-world applications.
